# Haplotype Map of Sickle Cell Anemia in Tunisia

**DOI:** 10.1155/2014/938301

**Published:** 2014-07-02

**Authors:** Imen Moumni, Maha Ben Mustapha, Sarra Sassi, Amine Zorai, Ikbel Ben Mansour, Kais Douzi, Dorra Chouachi, Fethi Mellouli, Mohamed Bejaoui, Salem Abbes

**Affiliations:** ^1^Laboratory of Molecular and Cellular Hematology, Pasteur Institute of Tunis, El Belvedere, 1002 Tunis, Tunisia; ^2^Service d'Immuno-Hématologie Pédiatrique, Centre National de Greffe de Moelle Osseuse, Tunis, Tunisia

## Abstract

*β*-Globin haplotypes are important to establish the ethnic origin and predict the clinical development of sickle cell disease patients (SCD). To determine the chromosomal background of *β* ^*S*^ Tunisian sickle cell patients, in this first study in Tunisia, we have explored four polymorphic regions of *β*-globin cluster on chromosome 11. It is the 5′ region of *β*-*LCR-HS2* site, the intervening sequence II (IVSII) region of two fetal (^G^ *γ* and ^A^ *γ*) genes and the 5′ region of *β*-*globin* gene. The results reveal a high molecular diversity of a microsatellite configuration describing the sequences haplotypes. The linkage disequilibrium analysis showed various haplotype combinations giving 22 “extended haplotypes”. These results confirm the utility of the *β*-globin haplotypes for population studies and contribute to knowledge of the Tunisian gene pool, as well as establishing the role of genetic markers in physiopathology of SCD.

## 1. Introduction

The haplotype of the *β* *-globin* gene cluster located on chromosome 11 has been used widely to obtain information about human variation, genetic relationship, and evolutionary analysis. The *β* ^*S*^ gene responsible for sickle cell disease (SCD) [*β*6(A3)Glu→Val, G*A*G→G*T*G] [1] has been found to be associated with five different restrictions haplotypes (HR). These haplotypes are designated the Benin (BEN), Bantu, or Central African Republic (BAN or CAR), Senegal (SEN), Cameroon (CAM), and Arab-Indian (ARB) types, according to the geographical area in which they are most commonly found [2–6]. Such a geographic prevalence of the *β* ^*S*^ gene associated with specific haplotypes has been argued to demonstrate the independent origin of the *β* ^*S*^ mutation in these regions [5, 7]. This assumption has been rejected by others who uphold the fact that the origin of the *β* ^*S*^ mutation is unicentric [8].

However, historically, *β*-globin haplotypes were established by Kan and Dozy [9], using restriction fragment length polymorphism (RFLP) analysis across the *β*-globin cluster. Previous attempts to correlate these haplotypes as predictors of clinical phenotypes observed in SCD have not been successful. We speculate that the distribution and number of RFLP sites used historically to define *β*-haplotypes are not sufficient to define the full range of genetic variations in this region. To test our hypothesis, we performed a polymorphism genotyping framework analysis across the *β*-globin cluster.

In the present study, we report the molecular investigations of four repeats sequences configurations (AT)_*x*_N_12_(AT)_*y*_ motif within the 5′* HS2* region of *β* *-LCR* site, (TG)_*n*_(CG)_*m*_ motif within IVSII region of fetal globin gene (^G^ *γ* and ^A^ *γ*), and (AT)_*x*_T_*y*_ motif within 5′ region of *β* *-globin* gene region of Tunisian *β* ^*S*^ chromosomes with five different RFLP-haplotypes (HR) described previously by Imen et al. [10]. Besides, we have searched an association between these genetic markers in order to determine a specificity for the Tunisian *β* ^*S*^ chromosome. Indeed, the “extended haplotype” (HE), regrouping RFLP and sequence haplotype (HS), is present in each of the ethnic groups as specific to *β* ^*S*^ chromosome and could be involved in the phenotypic expression of the disease.

## 2. Patients and Methods

### 2.1. Patients

The study was performed on 242 no consanguineous sickle cell patients from the Pediatrics services of the university hospital of Tunis. Blood samples were collected in EDTA as anticoagulant. All the patients were from Tunisia. Mean patient age was 13.17 years. The homozygous SS state was confirmed by family studies and in some cases by the direct detection of the *β* ^*S*^ mutation using the restriction enzyme* DdeI*.

### 2.2. DNA Sequence Analysis

**Figure 1:**
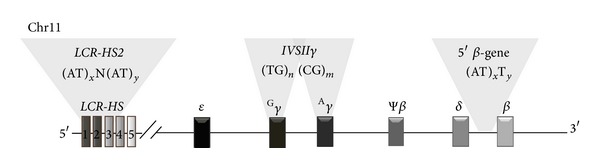
Map of the *β* *-globin* gene cluster and the polymorphic sites analyzed for sequence haplotypes.

Genomic DNA was isolated from peripheral blood leukocytes by phenol-chloroform extraction method [11]. The following framework polymorphisms were investigated by polymerase chain reaction (PCR) and direct sequencing: (AT)_*x*_N_12_(AT)_*y*_ repeat configurations within the 5′* HS2* region of *β* *-LCR* site [12, 13], (TG)_*n*_ (CG)_*m*_ configurations in the* IVSII* region of fetal globin gene (^G^ *γ* and ^A^ *γ*) [14], and (AT)_*x*_T_*y*_ repeat configuration in the 5′ region of *β* *-globin* gene [15] (Figure 1), using respective couples of primers as summarized in Table 1. Tunisian *β* ^*S*^ RFLP haplotypes (HR) described previously in Imen et al. have been used in this study [10].

### 2.3. Statistical Analysis

Given some of our genotypes that have an unknown gametic phase and include a large number of alleles, Arlequin, a program for the analysis of population genetic data, was used to perform a likelihood method for the analysis of linkage disequilibrium between the genetic marker configurations in each chromosome. Statistical significance was set at *P* < 0.05 [16].

The relationship between restriction haplotype and genetic markers was investigated by the uses of a PCA (principal component analysis) analysis. This analysis reduces a large number of variables to a few orthogonal variables called principal components (PC), which describe the largest covariance in the data analyzed as allele frequencies [17].

## 3. Results

### 3.1. Framework Analysis

**Figure 2:**
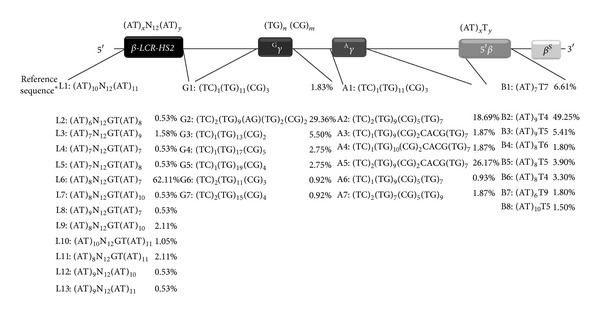
Molecular sequences and allele frequencies of the microsatellite configurations of *β* *-LCR-HS2* region,* IVSII* of fetal gene (^G^ *γ* and ^A^ *γ*), and 5′ of *β* *-globin* gene region. The configurations of *β* *-LCR-HS2* were named from L1 to L13, those of* IVSII-* ^G^ *γ* were named from G1 to G7, those of* IVSII-* ^A^ *γ* were named between A1 and A7, and those 5′ of *β* *-globin* gene were named from B1 to B8. L1, G1, A1, and B1 are the reference sequence configurations from the HUMHBB*. *The nucleotides are numbered according to the HUMHBB 73308 bp GenBank, Version: U01317.1 GI:455025.

Molecular data and allele frequencies concerning the microsatellite configurations, observed in *β* ^*S*^ chromosomes, are described in Figure 2. The (AT)_*x*_N_12_(AT)_*y*_ configurations of 5′* HS2* region of *β* *-LCR* were named from L1 to L13, the (TG)_*n*_ (CG)_*m*_ configurations of* IVSII* region of ^G^ *γ* gene were named from G1 to G7, the (TG)_*n*_ (CG)_*m*_ configurations of* IVSII* region of ^A^ *γ* gene were named from A1 to A7, and the (AT)_*x*_T_*y*_ configurations 5′ region of *β* *-globin* gene were named from B1 to B8.

### 3.2. (AT)_*x*_N_12_(AT)_*y*_ Motif in 5′* HS2* Region of *β* *-LCR* Site

In this study, we used the results previously published in the article by Ben Mustapha et al. [13]. Figure 2 shows the existence of several variations in the 5′*β* *-LCR HS-2* for *β* ^*S*^ chromosomes and the L6 (AT)_8_N_12_GT(AT)_7_ configuration was predominant in the studied sickle cell disease population with 62.11% of the total alleles.

### 3.3. (TG)_*n*_ (CG)_*m*_ Motif in IVSII Region of Fetal Globin (^G^ *γ* and ^A^ *γ*) Gene

Seven different microsatellite configurations of the (TG)_*n*_ (CG)_*m*_ motif were found among *β* ^*S*^ chromosomes in the* IVSII* region of ^G^ *γ-globin* gene (Figure 2). One novel sequence configurations G2 (TC)_2_(TG)_9_(AG)(TG)_2_(CG)_2_ was found and it was predominant with 29.36% of total alleles. In the* IVSII* region of ^A^ *γ-globin* gene, seven different microsatellite configurations of the (TG)_*n*_ (CG)_*m*_ motif were found among Tunisian *β* ^*S*^ chromosomes (Figure 2). Two novel configurations A3 (TC)_1_(TG)_9_(CG)_2_CACG(TG)_7_ and A5 (TC)_2_(TG)_9_(CG)_2_CACG(TG)_7_ were found and the A5 was predominant with 26.17% of total alleles which shows specificity to Tunisian *β* ^*S*^ chromosomes. The (AC)_1_(TG)_11_ (CG)_3_ sequence as a reference configuration correlates with a normal chromosome in both ^G^ *γ*- and ^A^ *γ*-*globin* genes named G1 and A1.

### 3.4. (AT)_*x*_T_*y*_ Motif in 5′ Region of *β* *-Globin* Gene

Several microsatellite configurations of the (AT)_*x*_T_*y*_ motif in the 5′*β*-*globin* gene studied among *β* ^*S*^ chromosomes were shown. The most frequent configuration was B2 (AT)_9_T_4_, it is specific to Tunisian *β* ^*S*^ chromosomes with an allele frequency of 49.25% (Figure 2).

### 3.5. Marker Combinations

#### 3.5.1. Arlequin Results

**Figure 3:**
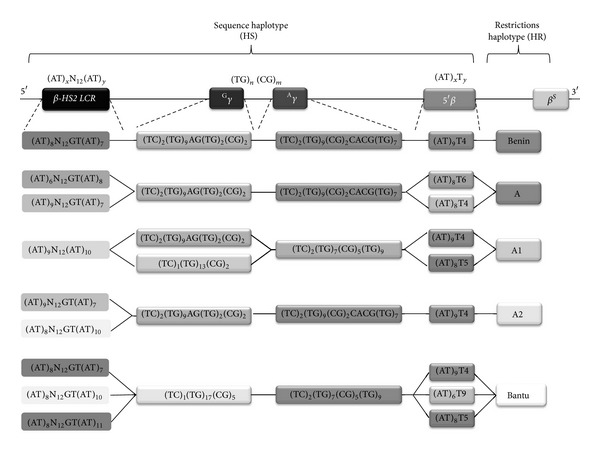
“Extended haplotype” (HE) in the *β*-globin locus performed first by “ARLEQUIN” and secondly by principal component analysis.

The Arlequin results, by the linkage disequilibrium test between genetic markers, showed an association between microsatellites motifs in regions of 5′* HS2 *β*-LCR*,* IVSII* of fetal gene (^G^ *γ* and ^A^ *γ*) and 5′ of *β* *-globin* gene defining a sequences haplotypes (HS). However, the data confirmed that the HR is in strict linkage disequilibrium with HS. Indeed, each association constitutes a haplotype which appears to be specific to Tunisian *β* ^*S*^ chromosome named “extended haplotype” (HE). The extended haplotype designates possible combinations obtained by the linkage disequilibrium test; it was performed first by “ARLEQUIN” and afterwards by PCA. The results of possible combinations grouping the HR and HS, summarized in Table 2, show 22 “extended haplotypes.” In fact, the Benin HR was associated with the configuration L6 in 5′*β*-*LCR-HS2*, G2 in* IVSII* ^G^ *γ-globin* gene, A5 in* IVSII* ^A^ *γ*-*globin* gene, and B2 in 5′*β*-*globin* gene (Figure 3).

#### 3.5.2. PCA Results

**Figure 4:**
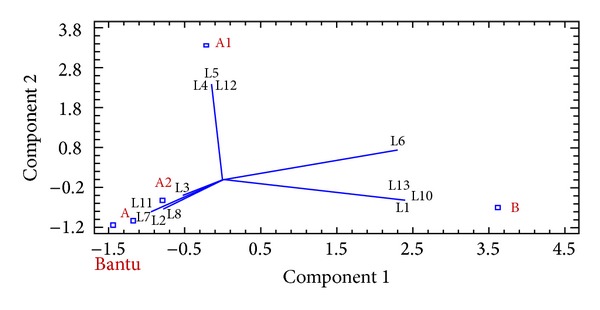
Correlation between restrictions haplotypes (HR) [B, A1, A2, A and Bantu] and microsatellites configurations of 5′* HS2 *β*-LCR* region [L1 to L13*] by PCA analysis (PC1+2 = 65,720%). *The microsatellite configurations are designated according to Figure 2.

**Figure 5:**
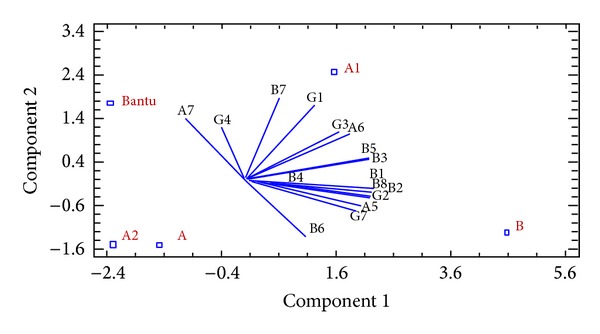
Correlation between restrictions haplotypes (HR) [B, A1, A2, A and Bantu] and sequence haplotype (HS) of following polymorphic region:* IVSII***** ^G^ *γ-globin* gene [G1 to G7*],* IVSII* ^A^ *γ-globin* gene [A1 to A7*], and 5′*β* *-globin* gene [B1 to B8*] by PCA analysis (PC1+2 = 80,521%). *The microsatellite configurations are designated according to Figure 2.

In order to verify and complete our Arlequin study, we used the PCA analysis. It was applied to visualize the distribution of polymorphic regions according to restriction haplotypes (HR) of SCD individuals and to achieve an adequate condensation of the information. It was performed to establish an overview about the correlation between the HR and the polymorphic regions, namely, (AT)_*x*_N_12_(AT)_*y*_ microsatellite configurations of 5′*β*-*LCR HS2* region, (TG)_*n*_ (CG)_*m*_ configurations of* IVSII* region of fetal genes (^G^ *γ* and ^A^ *γ*), and (AT)_*x*_T_*y*_ configurations of 5′ region of *β* *-globin* gene (Figures 4 and 5).

The linkage disequilibrium test was performed by “ARLEQUIN” software (Table 2) after being performed by PCA (Figure 3) to deduce the significance as *P* value. Probability values of *P* < 0.05 were considered to be statistically significant.

The data revealed that about 66% of the total variation could be explained by the first two PCs in Figure 4. Additionally, the PCA in Figure 5 revealed that the two first PCs together accounted for 80% of the total observed variability (data not shown) according to “Statgraphics plus 5.0” software.

Results, from the PCA of Figure 4, indicate that Benin HR (B) is shown to be near to L1, L6, L10, and L13 configurations of *β* *-LCR HS2* region. Figure 5 shows that B haplotype is additionally near to G2 and G7 configurations of* IVSII-* ^G^ *γ* region, to A5 configuration of* IVSII-* ^A^ *γ* region, and to B1, B2, B3, B4, B5, and B6 configurations of 5′*β* *-globin* gene. Thus, B haplotype found close to these configurations shows a positive correlation. Afterwards, the linkage disequilibrium test was performed to deduce a unique and important association between Benin and microsatellites configurations defining a combined haplotype, named the “extended haplotype”: “L6-G2-A5-B2” (Figure 3).

Analyzing Figures 4 and 5, we observe that A1 haplotype is near to L4, L5, and L12 configurations of *β* *-LCR HS2* region, G1 and G3 of* IVSII-* ^G^ *γ* region, A6 of* IVSII-* ^A^ *γ*, and B7 configuration of 5′*β*-*globin* gene. Table 2 of “ARLEQUIN” test shows that haplotype A1 could be associated with the following (HS): L12-G3 or G2-A6-B5 or B2 or B7 or B8. However, the linkage disequilibrium test according to “ARLEQUIN” data and PCA correlations shows four possible combinations between A1 and HS; the deduced “extended haplotypes” are L12-G3-A6-B2; L12-G3-A6-B5; L12-G2-A6-B2; and L12-G2-A6-B5 (Figure 3).

The A, A2, and Bantu haplotypes which are atypical ones are shown to be too near to one another referring to PCA results (Figure 4), since some configurations are in common such as L2, L3, L7, L8, and L11 of *β* *-LCR HS2* region.

Furthermore, Bantu haplotype is relatively near to G4 and A7 configurations of* IVSII* regions of fetal gene (^G^ *γ* and ^A^ *γ*), respectively, while no configurations of these regions, mentioned above, are shown to be associated with A and A2 haplotypes.

Be noted that we have observed, in PCA analysis, the correlation with only one haplotype, while several configurations of polymorphic regions have been displayed to be associated with other haplotypes according to the significant *P* value (*P* < 0.005), such as G2 configuration which seems to be associated with only B haplotype (Figure 5), whereas considering *P* value, it will be also associated with atypical haplotypes A (*P* = 0.0050), A1 (*P* = 0.0373), and A2 (*P* = 0.0405).

Additionally, it is important to mention that some microsatellite configurations, present initially in PCA plots (Figures 4 and 5), are not shown in Table 2, because of *P* value which is lower than 0.005 (*P* < 0.005) with studied restriction haplotypes, so the association is insignificant.

According to the linkage disequilibrium test (Figure 3), some correlations could be illustrated here, between A haplotype and HS, namely, the HE: L2 or L8-G2-A5-B4 or B6, between A2 haplotype and HS, namely, the HE: L9 or L8-G2-A5-B2, and between Bantu haplotype and HS, namely, the HE: L6 or La or L11-G4-A7-B2 or B5 or B7.

**Figure 6:**
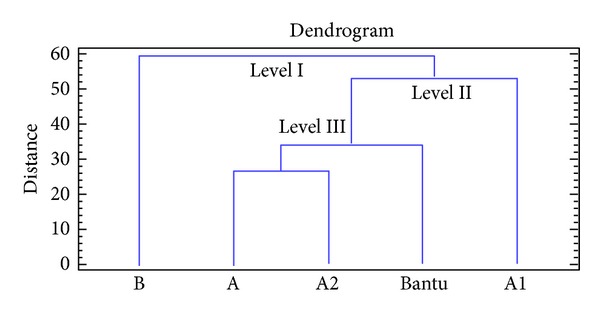
Dendrogram constructed by the neighbour joining method reflecting the relationships between restrictions haplotypes (HR) and sequence haplotypes (HS) based on PCA analysis.

Afterwards, the genomic relationship between the studied haplotypes was presented in a dendrogram according to PCA data (Figure 6) to verify previous observations and suggestions in order to confirm the accordance with our hypothesis that is published in our previous publication Imen et al. [10].

The neighbor-joining tree in Figure 6 was constructed according to maximum likelihood of the microsatellites configurations at *β*-globin locus* in *5′*β*-*HS2 LCR* region,* IVSII* region of ^(G and A)^ *γ-globin* gene, and 5′region of *β*-*globin* gene among five restrictions haplotypes. This dendrogram showed three main levels. The first one highlights that B haplotype is the farthest one (distance of B = 60) compared to the others (distance of A and A2 = 28, Bantu = 36, and A1 = 54), hence so to conclude that B haplotype is the ancestral haplotype. Through the second level, we found that A1 haplotype could be the common ancestor of A, A2, and Bantu haplotypes, since it appears upon this group while it is the farthest one. The third level shows that A and A2 haplotypes diversified from the same ancestor, the Bantu haplotype, and that the genomic distance between these haplotypes is very close and their ramifications are short. Based on these findings, we can suggest that these haplotypes are outcome of crossing over.

## 4. Discussion

There are five major *β* ^*S*^-globin cluster haplotypes in the world: four in Africa (Senegal, Benin, Bantu or CAR, and Cameroon) and one in Asia (Arabian-Indian). The Benin haplotype has been reported to be associated with *β* ^*S*^ genes in Algeria, Tunisia, Egypt, Jordan, Lebanon, Sicily, Greece, and Turkey [18–21], confirming a common genetic background for all the North African *β* ^*S*^ alleles. Historic data from these countries indicate that the Benin *β* ^*S*^ gene has traveled from Central West Africa to North Africa and various Mediterranean countries [3].

In Tunisia, sickle cell anemia haplotype was reported for the first time by Abbes et al. [20] and later by Imen et al. [10] describing six haplotypes: Benin is the most common, Bantu, and four atypical haplotypes [10].

Previously, RFLP analysis has been used as the common approach to establish *β*-haplotypes. Thus, our prior study [10] showed that the *β* ^*S*^ chromosome background, characterized by several atypical haplotypes, suggests that the restriction sites are not equally informative to know its origin. Indeed, we need further analysis focusing on other polymorphisms, namely, the microsatellites configurations linked to the *β*-globin cluster, which defines the sequence haplotypes (HS).

The sequencing results revealed a great molecular heterogeneity of microsatellites repetitions with the predominance of some motifs which are specific to *β* ^*S*^ chromosome such as the following configurations L6 (AT)_8_N_12_GT(AT)_7_ within the 5′* HS2* region of *β* *-LCR* site, the two newest configurations G2 (TC)(TG)_9_(AG)(TG)_2_(CG)_2_ and A5 (TC)_2_(TG)_9_(CG)_2_CACG(TG)_7_ within* IVSII* region of the fetal globin genes (^G^ *γ* and ^A^ *γ*), respectively, and the configuration (AT)_9_T_4_ with the 5′ region of *β* *-globin* gene, confirming the diversity of Tunisian *β* ^*S*^ chromosome.

Therefore, our findings demonstrate a great molecular diversity of sequences haplotypes (HS) and specificity to Tunisian *β* ^*S*^ chromosomes. Furthermore, to study the origin of ethnic groups, we need to correlate the HS with HR. Since the HS is represented by a major microsatellite configuration. Here we describe a discordance between the HR and the HS; the HS alone cannot establish the genetic map of sickle cell anemia in Tunisia and to describe the genetic structure of the Tunisian population, therefore, we must study the “extended haplotype” which is obtained by a combination between HS and HR.

Our data reveals that the motifs L6 (AT)_8_N_12_GT(AT)_7_, G2 (TC)(TG)_9_(AG)(TG)_2_(CG)_2_, A5 (TC)_2_(TG)_9_(CG)_2_CACG(TG)_7_, and B2 (AT)_9_T_4_ are closely associated with (B) Benin restriction haplotype. The results different from those observed among other studies [21, 22] demonstrate a new genetic identity of the Benin Tunisian sickle cell chromosome.

The atypical haplotypes such as A and A2 haplotypes were associated with several motifs with common sequences: L3, L2, L7, L8, and L11 in the 5′* HS2* region of *β* *-LCR* site and with B2, B4, and B6 in the 5′region of *β* *-globin* gene. Also, the (A1) haplotype is associated with L12 in 5′* HS2 *β*-LCR*, with G1 and G3 in the* IVSII* ^G^ *γ* gene, and with B2 and B5 in 5′*β*-*globin* gene. These results suggest that such specific genetic profiles may result from a crossing over at the Hot-Spot region between the *β* *-LCR* site and *β* *-globin* gene.

In fact, it has been demonstrated that a microsatellite sequences can appear in association with different haplotypes. The same microsatellite could be associated with specific haplotype. In general, involvement of the same polymorphism with more than one haplotype is most consistent with the crossing over of 5′ into the *β*-globin cluster, presumably within the region of increased recombination, the Hot-Spot region.

Furthermore, the sequences L10 (AT)_10_N_12_GT(AT)_11_, G2 (TC)(TG)_9_(AG)(TG)_2_(CG)_2_, A2 (TC)_2_(TG)_9_(CG)_5_(TG)_7_, and B4 (AT)_8_T_6_ motifs were strongly associated with the atypical haplotype (A). These data exclude the suggestion of recombination in Hot-Spot region and confirm that the (A) haplotype is specific of Tunisian chromosome *β* ^*S*^.

The PCA correlation plots show that A, A2, and Bantu haplotypes were associated with several and common configurations, unlike A1 and B haplotypes which seem to be independent, thus to suggest that these two latter haplotypes are specific to Tunisian SCD chromosome and not resulting from crossing over such as A, A2, and Bantu haplotypes.

The neighbor-joining tree was constructed according to maximum likelihood of the microsatellites configurations at *β*-globin locus* in *5′*β*-*HS2 LCR* region,* IVSII* region of ^(G and A)^ *γ-globin* gene, and 5′* region of *β*-globin* gene among five restrictions haplotypes. The results reveal that the (B) haplotype is the common ancestral restriction haplotype, the A1 is the ancestor of A, A2, and Bantu haplotypes, and the two haplotypes (A and A2) diversified from the same ancestor Bantu, suggesting that these haplotypes are outcome of crossing over. This finding comes to confirm our suggestion cited in the article previously published by Imen et al. [10].

In conclusion, our observations demonstrate that the traditional RFLP approach does not accurately reflect the complexity of the *β*-locus. Our study is the first detailed genomic analysis of this region conducted on Tunisian patients using modern genomics techniques. The results suggest that microsatellite mapping is not a better approach for defining *β*-haplotypes in our Tunisian population, but which can be used to correlate with the different clinical phenotypes observed in SCD and may have implications for basic research studies in globin gene regulation.

In the future, we intend to perform multifactorial analysis, namely, modifier gene analysis, of the genetic background of sickle cell chromosome, in order to improve our understanding of the natural history of sickle cell disease in SS patients. Our data also represents the first study in identification of the *β*-haplotype distribution in Tunisian patients, which may be useful for the clinical handling and outcome of the sickle cell patients.

## Figures and Tables

**Table 1:** Sequences of the primers used in different polymerase chain reaction protocols.

Region	Sequence of primers 5′ → 3′	Size pb
β-*LCR-HS2*	TAA GCT TCA GTT TTT CCT TAG T	740
(AT)_*x*_N_12_(AT)_*y*_	TAG ATC TGA CCC CGT ATG TGA GCA T	

IVSII ^G^γ	TGC TGC TAA TGC TTC ATT ACA A	782
(TG)_*n*_ (CG)_*m*_	AAG TGT GGA GTG TGC ACA TGA	

IVSII ^A^γ	TGC TGC TAA TGC TTC ATT ACA A	762
(TG)_*n*_ (CG)_*m*_	TAA ATG AGG AGC ATG CAC ACA C	

5′β-*Globin* gene	CGC TGA CCT CAT AAA TGC T	859
(AT)_*x*_T_*y*_	CTA CCA TAA TTC AGC TTT GGG A	

**Table 2:** Arlequin test for linkage disequilibrium between RFLP and sequence haplotypes. The microsatellite configurations are designated according to Figure 2.

RFLP haplotypes [10]	Sequence haplotypes			
	*β*-LCR-HS2 (AT)_*x*_N_12_(AT)_*y*_	IVSII ^G^ *γ* (TG)_*n*_ (CG)_*m*_	IVSII ^A^ *γ* (TG)_*n*_ (CG)_*m*_	5′*β* (AT)_*x*_T_*y*_
B	L6	G2	A5	B2

A	L2	G2	A5	B4
	L8			B6

A1	L12	G3	A6	B5 B2
		G2		B7 B8

A2	L9	G2	A5	B2
	L8			

Bantu	L6	G4	A7	B2
	L7			B5
	L11			B7
